# Factors Associated with Health-Seeking Behavior in Indonesia: Evidence from the Indonesian Family Life Survey 2014

**DOI:** 10.3390/medicina60101607

**Published:** 2024-10-01

**Authors:** Indah Laily Hilmi, Sofa D. Alfian, Rizky Abdulah, Irma Melyani Puspitasari

**Affiliations:** 1 Department of Pharmacology and Clinical Pharmacy, Faculty of Pharmacy, Universitas Padjadjaran, Sumedang 45363, West Java, Indonesia; indah22003@mail.unpad.ac.id (I.L.H.); sofa.alfian@unpad.ac.id (S.D.A.); r.abdulah@unpad.ac.id (R.A.); 2 Department of Pharmacy, Faculty of Health Science, Universitas Singaperbangsa Karawang, Karawang 41361, West Java, Indonesia; 3 Center of Excellence in Higher Education for Pharmaceutical Care Innovation, Universitas Padjadjaran, Sumedang 45363, West Java, Indonesia

**Keywords:** IFLS, health-seeking behavior, Indonesia, formal, chronic diseases

## Abstract

*Background and Objectives*: Health-seeking behavior is a critical determinant of health outcomes, particularly in countries like Indonesia. Given the increasing burden of noncommunicable diseases, understanding the factors that influence health-seeking behavior in this context is essential for developing more accessible and effective public health strategies. This study aimed to identify various factors associated with health-seeking behavior among patients with chronic diseases across Indonesia, especially in formal facilities. *Materials and Methods*: This study used a cross-sectional research design, utilizing Indonesian Family Life Survey (IFLS)-5 data. The inclusion criteria included respondents aged 20–74 years old with at least one chronic disease based on self-reported data. Respondents who did not receive treatment, practiced self-medication, or provided incomplete data were excluded. We used multivariate logistic regression to identify factors associated with health-seeking behavior in formal facilities. *Results*: The results revealed that 80.7% (*n* = 1993) of the 2471 respondents sought treatment in formal facilities, whereas 19.3% (*n* = 478) opted for informal facilities. Respondents who were Bugis (OR 9.187, 95% CI 2.182–38.683; *p* = 0.002), retired (OR 2.966, 95% CI 1.233–7.135; *p* = 0.015), did not smoke (OR 1.604, 95% CI 1.126–2.285; *p* = 0.009), made less than IDR 1,500,000 a month (OR 1.466, 95% CI 1.174–1.831; *p* = 0.000), had to travel more than 3 km to reach a treatment facility (OR 1.847, 95% CI 1.41–2.42; *p* = 0.000), or had more than one comorbidity (OR 1.396, 95% CI; *p* = 0.01) were more likely to seek treatment at formal facilities. *Conclusions*: These findings are expected to provide recommendations for policymakers, healthcare providers, and researchers to contribute to the development of targeted interventions that can improve healthcare access and utilization, ultimately enhancing health outcomes and equity in Indonesia.

## 1. Introduction

Health-seeking behavior is a critical determinant of health outcomes, particularly in a diverse and populous country such as Indonesia [1]. As an archipelago comprising over 16,000 islands and home to more than 278 million people across 38 provinces [2], Indonesia presents a unique and complex landscape for healthcare delivery and utilization [3]. The Indonesian healthcare system encompasses both formal and informal sectors, with public and private healthcare providers offering a range of services and traditional healers and self-medication practices playing a significant role in the informal sector [1].

Given the increasing burden of chronic diseases [4], understanding the factors that influence the choice between formal and informal healthcare facilities is essential for developing more accessible and effective public health strategies [1]. Noncommunicable diseases require regular medical check-ups, early diagnosis, and long-term management [5], making it imperative to address barriers to healthcare access. Improving access to health services is critical not only for managing these chronic conditions but also for enhancing overall health outcomes. Increased access can lead to increased health equity and promote cost-effectiveness because of the early management of health problems [4,6].

In 1968, Andersen proposed the Model of Utilization of Health Services, which posits that a person’s use of health services is influenced by three main factors: predisposing factors—such as sociodemographic characteristics; enabling factors—such as accessibility, financial capability, and perceived self-efficacy; and the need for care factors—including self-reported health status, chronic conditions, and perceived risk [7,8,9]. Recent studies have confirmed that lower education and cultural beliefs can significantly impact health-seeking behavior, with some communities preferring traditional healers over modern medical practitioners because of deeply rooted cultural norms and trust in traditional medicine [1,10,11]. Additionally, financial constraints, a lack of health insurance, and the accessibility and availability of healthcare services often deter individuals from seeking formal healthcare services [1,11,12,13,14]. The perceived quality of care, including the behavior of healthcare providers and the effectiveness of treatments, also influences individuals’ decisions to utilize healthcare services [14,15].

Despite the extensive research on healthcare preference for formal and informal facilities in various countries [16,17,18], there remains a significant research gap in understanding the specific factors influencing the choice between formal and informal healthcare facilities in Indonesia. While some studies have explored general healthcare utilization patterns [19], a comprehensive analysis integrating socioeconomic, cultural, and systemic factors unique to Indonesia is limited.

The Indonesian Family Life Survey (IFLS) was first conducted in 1993 and in 1997, 2000, 2007, and 2014 [20]. The IFLS aims to provide an overview of the health behaviors and outcomes of the general population of Indonesia and, as such, contains information on health service use, physical health, mental health, health behaviors, and important sociodemographic characteristics, including household assets [20]. The IFLS provides comprehensive data on individual and household characteristics, health status, and healthcare utilization in Indonesia, offering valuable insights into the determinants of health-seeking behavior. By leveraging the rich dataset provided by the IFLS 2014, a more complete version of previous IFLSs, this study aims to assess the various factors associated with the preference for formal and informal healthcare facilities in Indonesia, with a particular focus on formal health facilities. The findings from this study will provide a valuable reference for policymakers, healthcare providers, and researchers and are expected to contribute to the development of targeted interventions that can improve healthcare access and utilization, ultimately enhancing health outcomes and equity in Indonesia.

## 2. Materials and Methods

### 2.1. Design and Source of Data

The study applied a cross-sectional research design using data from the Indonesian Family Life Survey-5 (IFLS-5), which was conducted in 2014 and 2015. The RAND Corporation conducted the IFLS in collaboration with the Demographic Institute of the University of Indonesia, the University of California, Los Angeles (UCLA) in the United States, and the Centre for Population and Policy Studies at Gadjah Mada University. The IFLS-5 survey conducted from 2014 to 2015 collected data from approximately 83% of the population living in 13 provinces in Indonesia, encompassing over 30,000 people. The collected data were obtained from respondents selected through stratified sampling based on geographical location, followed by stratification based on households from the previous IFLS. [20]. More information about the IFLS study design can be obtained from http://www.rand.org/labor/FLS/IFLS.html (accessed on 17 August 2023). The IFLS survey and its procedures were reviewed and approved by the RAND’s Human Subjects Protection Committee (RAND’s IRB) code of approval: s0064-06-01-CR01 [20]. In addition, because this study used anonymous data from the IFLS, the research ethics committee of Universitas Padjadjaran Indonesia, waived the ethical approval with code of approval: 1242/UN6.KEP/EC/2023 and date of approval: 5 October 2023.

### 2.2. Inclusion and Exclusion Criteria

The inclusion criteria were respondents aged 20–74 years old and who had at least one chronic disease based on self-reported data. The exclusion criteria were respondents without treatment, practicing self-medication, or with incomplete data.

### 2.3. Data Collection

The data collected focused on factors influencing health-seeking behavior, including predisposing, enabling, and need factors [9]. Predisposing factors included age, gender, ethnicity, occupation, education level, lifestyle, religion, marital status, and religiosity. The enabling factors encompassed support systems for health services, such as monthly income, insurance, accessibility, and healthcare costs. The need for care factors assessed illness severity, including overall health, the number of chronic diseases, and perceived susceptibility.

Health-seeking behavior was defined as treatment choices made in formal or informal settings when individuals experienced health problems. Formal settings included government and private hospitals, community health centers (Puskesmas), clinics, and the practices of doctors and health workers [21]. Informal settings involved traditional healers such as shamans, Chinese herbalists, masseurs, and acupuncturists. The respondents who did not select any of these options were considered either not seeking treatment or practicing self-medication.

### 2.4. Data Analysis

Descriptive statistics were used to summarize respondent characteristics. Multivariate logistic regression with manual backward elimination was used to determine odds ratios (ORs) with 95% confidence intervals (95% CIs). The factors included in the multivariate analysis were those with *p*-values less than 0.25 in the bivariate analysis. The reference categories for all the variables were as follows: age 20–30 years (Age), male (Gender), Java (Ethnicity), bed rest (Occupation), no education (Level of education), smoking (Lifestyle), Catholic (Religion), not married (Marital status), very religious (Religiosity), IDR 1,500,000–IDR 2,500,000 (Monthly income), no insurance (Insurance), <3 km (Accessibility of distance), above IDR 100,000 (Healthcare cost), and two chronic diseases (The number of chronic diseases). These reference categories were defined a priori and were determined purposively by researchers. Some variables used the first category, while others used the middle or last category in order to obtain risk factors (OR > 1). The final model was considered significant with *p*-values less than 0.05. All the statistical analyses were conducted using Stata software version 14.0 (StataCorp LLC, College Station, TX, USA).

## 3. Results

### 3.1. Data Extraction

**Figure 1:**
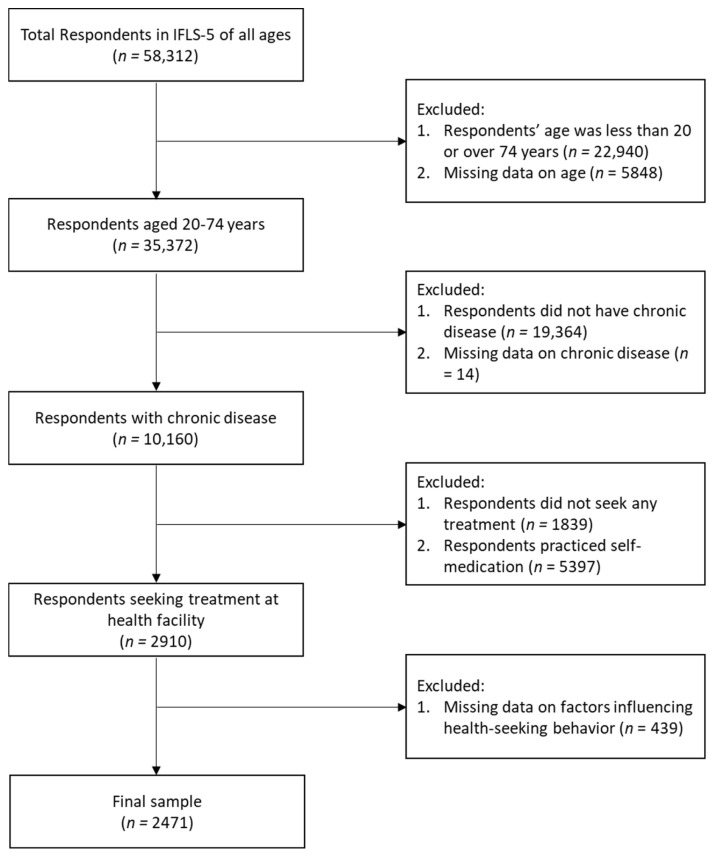
Diagram of participant selection from the IFLS-5.

As shown in Figure 1, 58,312 respondents were included in the IFLS-5 data. We included 35,372 respondents aged 20–74 years and excluded 22,940 respondents outside this age range. From the included group, we focused on 10,160 respondents who reported having at least one chronic disease, excluding 19,364 who had no chronic diseases and 5848 with missing data. Among the respondents with chronic diseases, 2910 individuals sought treatment at a health facility, whereas 7236 did not seek treatment at a health facility, 1839 did not seek any treatment, and 5397 resorted to self-medication. Additionally, 14 respondents had missing data. Among the 2910 respondents who sought treatment at a health facility, only 2471 had complete data.

### 3.2. Respondents’ Characteristics

The characteristics of the respondents are described in Table 1. Most of the respondents were 31–41 years old (24.3%), female (68.7%), Javanese (46.6%), with elementary school education (35.1%), Muslim (87.9%), had a job (59%), were married (81.7%), and chose formal facilities (80.7%).

### 3.3. Factors Influencing Health-Seeking Behavior

The factor that influenced health-seeking behavior among patients with chronic diseases in formal health facilities was ethnicity (*p*-value = 0.002), including Bugis (OR 9.187, 95% CI 2.182–38.683), followed by Sundanese (OR 3.946, 95% CI 2.601–5.985), Betawi (OR 2.279, 95% CI 1.233–4.248), Minang (OR 2.028, 95% CI 1.09–3.773), and Madura ethnicities (OR 0.285, 95% CI 0.166–0.489). Other factors were a retired occupation (OR 2.966, 95% CI 1.233–7.135; *p*-value = 0.015), having a nonsmoking habit (OR 1.604, 95% CI 1.126–2.285; *p*-value = 0.009), low income < IDR 1,500,000 (OR 1.624, 95% CI 1.108–2.378; *p*-value = 0.013), having insurance (OR 1.466, 95% CI 1.174–1.831; *p*-value = 0.000), accessibility to healthcare facilities more than 3 km (OR 1.847, 95% CI 1.410–2.420; *p*-value = 0.000), treatment costs below IDR 100,000 (OR 1. 964, 95% CI 1.526–2.529; *p*-value = 0.000), and having one comorbidity (OR 1.396, 95% CI 1.082–1.800; *p*-value = 0.010). The goodness-of-fit *p*-value of this model is 0.000, with an R-squared value of 0.103. Detailed information about the analyses is presented in Table 2 and Table S1 in the Supplementary Materials.

## 4. Discussion

This is the first study utilizing IFLS-5 data to examine factors influencing the preference between formal and informal healthcare facilities among patients with chronic diseases in Indonesia. This study identified eight factors influencing health-seeking behavior in formal health facilities, including predisposing factors (ethnicity, occupation, and lifestyle), enabling factors (income, insurance ownership, accessibility, and medical expenses), and need factors (the number of chronic diseases).

In terms of predisposing factors, ethnicity, occupation, and lifestyle were associated with choosing formal healthcare facilities. Bugis ethnicity was found to have a strong influence on healthcare facility preference (OR 9.187). While we noted that cultural practices and community support systems unique to certain ethnicities might also play a role, this study could not directly conclude this, unlike a previous study on health-seeking behavior among colorectal cancer patients in East Java, Indonesia [22]. Being retired was another significant factor (OR 2.966). This could be attributed to the availability of time, increased health awareness, and possibly better financial stability due to pensions or savings [23]. A study among type 2 diabetes mellitus patients in Central Java, Indonesia, also found that occupation could be associated with the decision to utilize formal healthcare facilities [24]. Individuals with nonsmoking habits were also more likely to choose formal healthcare facilities (OR 1.604). This aligns with the findings from Ismail et al. [25], who suggested that smokers tend to have a lower health consciousness [25]; therefore, they are less likely to seek formal healthcare services.

With respect to enabling factors, income, insurance ownership, accessibility, and treatment costs were associated with the preference for formal healthcare facilities. Low income was associated with increased formal health-seeking behavior (OR 1.624). This could be attributed to government subsidies or health insurance schemes targeting low-income groups. In addition, having insurance had a significant effect on the choice of formal healthcare facilities (OR 1.466). We used data from the survey conducted in 2014, when the National Health Insurance (JKN) program was introduced in Indonesia, replacing previous health insurance programs. Although insurance coverage was still expanding, the implementation of JKN for low-income families provided effective protection against healthcare expenses, thereby increasing access to formal health services [26,27]. This finding suggests that expanding insurance coverage could improve access to formal healthcare facilities. Similar findings have been reported in studies from Indonesia and Saudi Arabia, where insured individuals reported higher rates of seeking formal healthcare services [19,28]. Our study revealed that living more than 3 km away from a healthcare facility is associated with a greater likelihood of seeking formal healthcare (OR 1.847). Although a negative relationship between distance and health-seeking behavior is common [29], several contextual factors can explain this result. In Indonesia, primary health facilities (Puskesmas) are located in every subdistrict (kecamatan) and can often be more than 3 km from respondents’ homes. This distance might not be perceived as a barrier by those who need primary care. In addition, specialized healthcare facilities are often located farther away (e.g., in districts/kabupaten areas and provincial capitals), and individuals may travel longer distances for higher-quality care. Better transportation options for those living farther away might make it more feasible for them to access formal healthcare services [29]. Lower treatment costs (below IDR 100,000) were also associated with a higher formal healthcare facility preference (OR 1.964). Affordable treatment options make formal healthcare services more attractive. This finding is supported by a study in Vietnam that revealed that cost is a significant determinant of healthcare facility utilization [30].

The number of chronic diseases was the only need factor identified in this study. Individuals with one chronic disease were more likely to choose formal healthcare facilities, with odd ratios of 1.396. A study comparing visit rates among patients with chronic diseases to community health centers versus private practices in the United States also found that as the number of chronic conditions increases, the frequency or likelihood of seeking care might not rise proportionally [31]. This could be due to factors such as the complexity of managing multiple conditions, potential barriers to accessing care, or differing healthcare needs among those with multiple conditions. Managing multiple chronic diseases can be challenging and often requires more frequent visits to formal healthcare facilities [32]. This may deter individuals with multiple chronic diseases from utilizing formal care unless necessary.

Some factors, including age, gender, education level, religion, marital status, religiosity, overall health, and perceived susceptibility, were not significantly associated with healthcare facility preference in this study. These factors contrast with findings from studies in India and America [33,34]. Possible reasons for this could be the context or specific healthcare policies and interventions in place. For example, universal healthcare policies might minimize age-based differences in healthcare services [35], health–literacy programs and outreach efforts may reduce educational disparities [36], and inclusive practices in healthcare facilities are likely to respect and accommodate diverse religious backgrounds [37]. However, this study also revealed that there is an increasing trend of chronic disease in the age range of 31–40 years. This finding is consistent with the Indonesian basic health research data (Riskesdas) from 2013 to 2018. There is an increasing trend of several chronic diseases, including hypertension and diabetes mellitus, in all age groups, especially in young adults (25–34 years) and adults (35–44 years) [21,38].

This is the first study to use IFLS-5 data to examine the factors influencing the preference between formal and informal health facilities in Indonesia. This study provides a general overview of healthcare facility preference across a range of chronic diseases, such as hypertension, diabetes, tuberculosis, asthma, chronic pulmonary diseases, cardiovascular diseases, liver disorders, stroke, cancer, high cholesterol, prostate conditions, renal diseases, gastrointestinal disorders, mental health issues, and memory-related illnesses. It highlights that formal facilities are the preferred choice, chosen by 80.7% of respondents. This emphasizes the comparatively lower use of informal services. This finding contrasts with a study conducted in one Indonesian city, which reported that 64.9% of participants chose informal facilities, with herbal medicine (54.4%), ceragem (16.7%), and massage (12.3%) being the most common types [39].

The strengths of this study include its large sample size and the application of robust statistical methods, which increase the reliability and validity of the findings. Additionally, diverse demographic representations provide a comprehensive overview of the factors influencing healthcare facility preferences in Indonesia. However, there are limitations to consider. Potential biases in self-reported data could affect the accuracy of the results, and the cross-sectional nature of the study restricts the ability to draw causal inferences. Additionally, the exclusion of cases due to missing data may introduce some bias, as the characteristics of excluded participants could differ from those included, potentially impacting the generalizability of the findings. Furthermore, owing to the nature of the data, which represent a general population sample, the respondents’ characteristics in this study may not fully represent individuals with chronic diseases in Indonesia. Finally, this study may not account for all confounding variables, such as psychosocial factors, including cultural beliefs and self-efficacy, since we used secondary data.

Future research should investigate the longitudinal impact of these factors on healthcare facility preferences to understand how these influences evolve over time and potentially affect utilization patterns causally. Longitudinal studies can allow for observing changes and trends in healthcare facility choice, providing deeper insights into the effectiveness of interventions and the persistence of identified barriers. Additionally, future research should explore strategies for addressing missing data or conduct analyses to confirm that the exclusion due to missing data might not change the participants’ characteristics. Research could investigate healthcare facility choice among broader samples, which include individuals who are not seeking treatment in healthcare facilities. Furthermore, future research should focus on improving sample representativeness by targeting specific diseases in these populations. Research should also be conducted to assess other factors related to healthcare facility preference, such as knowledge, psychosocial factors, quality of service, severity staging, and the duration of disease [40,41,42].

The practical implications of this study highlight the need for targeted interventions to address these barriers. Strategies should focus on improving accessibility to formal healthcare services, particularly for those living more than 3 km away from facilities, and ensuring the affordability of treatments. Policymakers and healthcare providers should consider implementing mobile health clinics, telemedicine, and subsidized healthcare programs to reach underserved populations. Our findings also suggest that expanding insurance coverage could improve access to formal healthcare facilities. Increasing the awareness of formal healthcare facility utilization is also important. These efforts can lead to more equitable healthcare access and improved health outcomes for specific populations.

## 5. Conclusions

This study provides insights into how various factors influence people’s choices between formal and informal healthcare. Based on data from the Indonesian Family Life Survey (IFLS)-5, this study identified eight main factors that influence health-seeking behavior at formal facilities, including ethnicity, occupation, lifestyle, income, insurance ownership, accessibility, treatment costs, and the number of chronic diseases. The findings are expected to provide recommendations for policymakers, healthcare providers, and researchers to develop more targeted interventions to improve access to and the use of formal health services, thereby contributing to improved health outcomes and health equity in Indonesia.

## Figures and Tables

**Table 1:** Respondents’ characteristics (*n* = 2471).

Characteristics	Sample (*n*)	Proportion (%)
**Choice of medication**		
Informal	478	19.3
Formal	1993	80.7
**Age**		
20–30 years old	464	18.8
31–40 years old	601	24.3
41–50 years old	520	21
51–60 years old	509	20.6
>60 years old	377	15.3
**Gender**		
Female	1697	68.7
Male	774	31.3
**Ethnicity**		
Java	1152	46.6
Sundanese	364	14.7
Bali	155	6.3
Batak	117	4.7
Bugis	71	2.9
Tionghoa	11	0.4
Madura	65	2.6
Sasak	67	2.7
Minang	104	4.2
Banjar	77	3.1
Makasar	36	1.5
Nias	13	0.5
Palembang	15	0.6
Toraja	9	0.4
Betawi	103	4.2
Dayak	2	0.1
Melayu	27	1.1
Komering	10	0.4
Aceh	4	0.2
Other Sumbagsel	61	2.5
Banten	8	0.3
**Occupation**		
Work	1236	50
Job seeker	10	0.4
Study period	13	0.5
Homemaker	938	38
Pensions	92	3.7
Unemployed	72	2.9
Bed rest	110	4.5
**Level of education**		
No education	133	5.4
Elementary school	867	35.1
Junior high school	417	16.9
Senior high school	672	27.2
University	377	15.3
Others	5	0.2
**Lifestyle**		
Nonsmoking	1847	74.7
Smoking	624	25.3
**Religion**		
Islam	2172	87.9
Catholic	36	1.5
Protestant	102	4.1
Hinduism	161	6.5
**Marital status**		
Not married	140	5.7
Married	2019	81.7
Divorce	312	12.6
**Religiosity**		
Very religious	448	18.1
Religious	1536	62.2
Less religious	437	17.7
Not religious	50	2
**Monthly income**		
<IDR 1,500,000	1903	77
IDR 1,500,000–IDR 2,500,000	221	8.9
IDR 2,500,000–IDR 3,500,000	152	6.2
>IDR 3,500,000	195	7.9
**Insurance**		
No insurance	992	40.1
Have insurance	1479	59.9
**Accessibility of distance**		
<3 km	1829	74
>3 km	642	26
**Healthcare costs**		
Under IDR 100,000	1935	78.3
Above IDR 100,000	536	21.7
**Health status**		
Very healthy	193	7.8
Fairly healthy	1133	45.9
Less healthy	1058	42.8
Not healthy	87	3.5
**The number of chronic diseases**		
1 chronic disease	1481	59.9
2 chronic diseases	610	24.7
≥3 chronic diseases	380	15.4
**Perceived susceptibility**		
No	2096	84.8
Yes	375	15.2

**Table 2:** Multivariate associations with medical decisions on formal facilities.

Characteristics	OR [95% CI]	*p*-Value
**Age**		
20–30 years old	Reference	
31–40 years old	0.847 [0.599–1.198]	0.348
41–50 years old	0.838 [0.576–1.220]	0.356
51–60 years old	0.950 [0.627–1.439]	0.808
>60 years old	1.366 [0.838–2.226]	0.211
**Gender**		
Female	1.072 [0.740–1.1552]	0.714
Male	Reference	
**Ethnicity**		
Java	Reference	
Sundanese	3.946 [2.601–5.985]	0.000 **
Bali	2.330 [0.471–11.52]	0.300
Batak	0.092 [0.525–1.449]	0.708
Bugis	9.187 [2.182–38.683]	0.002 **
Tionghoa	0.792 [0.169–3.709]	0.767
Madura	0.285 [0.166–0.489]	0.000 **
Sasak	1.481 [0.743–2.952]	0.264
Minang	2.028 [1.090–3.773]	0.026 **
Banjar	0.958 [0.544–1.688]	0.883
Makasar	1.593 [0.593–4.276]	0.355
Nias	1.590 [0.366–6.913]	0.536
Palembang	0.890 [0.261–3.037]	0.853
Toraja	1.857 [0.21–16.438]	0.578
Betawi	2.279 [1.233–4.248]	0.010 **
Dayak	0.840 [0.048–14.731]	0.905
Melayu	2.279 [1.223–4.248]	0.182
Komering	0.984 [0.194–4.985]	0.985
Aceh	1.268 [0.119–13.474]	0.844
Other Sumbagsel	0.707 [0.390–1.284]	0.255
Banten	2.042 [0.240–17.400]	0.514
**Occupation**		
Work	1.457 [0.893–2.379]	0.132
Job seeker	0.850 [0.187–3.865]	0.833
Study period	1.079 [0.243–4.789]	0.920
Homemaker	1.458 [0.868–2.448]	0.154
Pensions	2.966 [1.233–7.135]	0.015 **
Unemployed	1.966 [0.641–2.991]	0.408
Bed rest	Reference	
**Level of education**		
No education	Reference	
Elementary school	0.780 [0.444–1.369]	0.386
Junior high school	0.959 [0.517–1.780]	0.894
Senior high school	0.733 [0.401–1.338]	0.311
University	0.9242 [0.491–1.807]	0.857
Others	0.455 [0.063–3.266]	0.434
**Lifestyle**		
Nonsmoking	1.604 [1.126–2.285]	0.009 **
Smoking	Reference	
**Religion**		
Islam	0.859 [0.328–2.253]	0.758
Catholic	Reference	
Protestant	0.712 [0.244–2.073]	0.533
Hinduism	0.806 [0.131–4.940]	0.815
**Marital status**		
Not married	Reference	
Married	1.178 [0.717–1.936]	0.518
Divorce	0.975 [0.531–1.790]	0.934
**Religiosity**		
Very religious	Reference	
Religious	0.967 [0.715–1.307]	0.826
Less religious	0.867 [0.600–1.259]	0.453
Not religious	1.546 [0.591–1.609]	0.314
**Monthly income**		
<IDR 1,500,000	1.624 [1.108–2.378]	0.013 **
IDR 1,500,000–IDR 2,500,000	Reference	
IDR 2,500,000–IDR 3,500,000	1.322 [0.781–2.239]	0.298
>IDR 3,500,000	1.067 [0.650–1.752]	0.798
**Insurance**		
No insurance	Reference	
Have insurance	1.466 [1.174–1.831]	0.000 **
**Accessibility of distance**		
<3 km	Reference	
>3 km	1.847 [1.410–2.420]	0.000 **
**Healthcare costs**		
Under IDR 100,000	1.964 [1.526–2.529]	0.000 **
Above IDR 100,000	Reference	
**The number of chronic diseases**		
1 chronic disease	1.396 [1.082–1.800]	0.010 **
2 chronic diseases	Reference	
≥3 chronic diseases	1.153 [ 0.816–1.628]	0.247

Sig ** *p*-value < 0.05; goodness-of-fit *p*-value of the final model: 0.000; pseudo-R-squared: 0.103.

## Data Availability

The data presented in this study are available in the manuscript and in the Supplementary Materials.

## Supplementary Materials

The following supporting information can be downloaded at https://www.mdpi.com/article/10.3390/medicina60101607/s1. Table S1: Bivariate Analysis Result.
