# Supplementary material for: Factors Associated with Health-Seeking Behavior in Indonesia: Evidence from the Indonesian Family Life Survey 2014
Source: Medicina (Kaunas). 2024 Oct 1;60(10):1607. doi: 10.3390/medicina60101607 (PMC11509421; doi:10.3390/medicina60101607)
Supplement: Supplementary file 1 [file medicina-60-01607-s001.zip › medicina-3150953-SI.pdf]

Table S1. Bivariate Analysis Result.

Characteristics	Informal Facilities		Formal Facilities		*p*-value
	n	%	n	%	
**Age**					0.031*
20–30 years old	87	18.8	377	81.3	
31–40 years old	128	21.3	473	78.7	
41–50 years old	112	21.5	408	78.5	
51–60 years old	99	19.4	410	80.6	
>60 years old	52	13.8	325	86.2	
**Gender**					0.000*
Female	293	17.3	1404	82.7	
Male	185	23.9	589	76.1	
**Ethnicity**					0.000*
Java	266	23.1	886	76.9	
Sundanese	29	8.0	335	92.0	
Bali	18	11.6	137	88.4	
Batak	35	29.9	82	70.1	
Bugis	2	2.8	69	97.2	
Tionghoa	3	27.3	8	72.7	
Madura	30	46.2	35	53.8	
Sasak	11	16.4	56	83.6	
Minang	13	12.5	91	87.5	
Banjar	19	24.7	58	75.3	
Makasar	5	13.9	31	86.1	
Nias	3	23.1	10	76.9	
Palembang	4	26.7	11	73.3	
Toraja	1	11.1	8	88.9	
Betawi	13	12.6	90	87.4	
Dayak	1	50.0	1	50.0	
Melayu	3	11.1	24	88.9	
Komering	2	20.0	8	80.0	
Aceh	1	25.0	3	75.0	
Other Sumbagsel	18	29.5	43	70.5	
Banten	1	12.5	7	87.5	
**Occupation**					0.002*
Work	262	21.2	974	78.8	
Job seeker	3	30.0	7	70.0	
Study period	3	23.1	10	76.9	
Homemaker	154	16.4	784	83.6	
Pensions	9	9.8	83	90.2	
Unemployed	15	20.8	57	79.2	
Bed rest	32	29.1	78	70.9	
**Level of Education**					0.093*
No education	18	13.5	115	86.5	
Elementary school	171	19.7	696	80.3	
Junior high school	70	16.8	347	83.2	
Senior high school	148	22.0	524	78.0	
University	69	18.3	308	81.7	
Others	2	40.0	3	60.0	

**Lifestyle**					0.000*
Nonsmoking	317	17.2	1530	82.8	
Smoking	161	25.8	463	74.2	
**Religion**					0.000*
Islam	424	19.5	1748	80.5	
Catholic	7	19.4	29	80.6	
Protestant	28	27.5	74	72.5	
Hinduism	19	11.8	142	88.2	
**Marital status**					0.217*
Not married	33	23.6	107	76.4	
Married	393	19.5	1626	80.5	
Divorce	52	16.7	260	83.3	
**Religiosity**					0.182*
Very Religious	79	17.6	369	82.4	
Religious	291	18.9	1245	81.1	
Less Religious	100	22.9	337	77.1	
Not Religious	8	16.0	42	84.0	
**Monthly income**					0.002*
< IDR 1,500,000	338	17.8	1565	82.2	
IDR 1,500,000–IDR 2,500,000	57	25.8	164	74.2	
IDR 2,500,000–IDR 3,500,000	33	21.7	119	78.3	
> IDR 3,500,000	50	25.6	145	74.4	
**Insurance**					0.000*
No Insurance	235	23.7	757	76.3	
Have Insurance	243	16.4	1236	83.6	
**Accessibility of Distance**					0.000*
< 3 kilometers	387	21.2	1442	78.8	
> 3 kilometers	91	14.2	551	85.8	
**Healthcare costs**					0.000*
Under IDR 100,000	332	17.2	1603	82.8	
Above IDR 100,000	146	27.2	390	72.8	
**Health status**					0.907
Very healthy	35	18.1	158	81.9	
Fairly healthy	215	19.0	918	81.0	
Less healthy	210	19.8	848	80.2	
Not healthy	18	20.7	69	79.3	
**The number of chronic diseases**					0.079*
1 chronic disease	267	18.0	1214	82.0	
2 chronic diseases	136	22.3	474	77.7	
≥ 3 chronic diseases	75	19.7	305	80.3	
**Perceived susceptibility**					0.939
Not	406	19.4	1690	80.6	
Yes	72	19.2	303	80.8	

*$p < 0.25$: entered into multivariate logistic regression
